# Induction of CD4^+^CD25^+^FOXP3^+^ Regulatory T Cells during Human Hookworm Infection Modulates Antigen-Mediated Lymphocyte Proliferation

**DOI:** 10.1371/journal.pntd.0001383

**Published:** 2011-11-08

**Authors:** Natasha Delaqua Ricci, Jacqueline Araújo Fiúza, Lilian Lacerda Bueno, Guilherme Grossi Lopes Cançado, Pedro Henrique Gazzinelli-Guimarães, Virgillio Gandra Martins, Leonardo Ferreira Matoso, Rodrigo Rodrigues Cambraia de Miranda, Stefan Michael Geiger, Rodrigo Correa-Oliveira, Andréa Gazzinelli, Daniella Castanheira Bartholomeu, Ricardo Toshio Fujiwara

**Affiliations:** 1 Department of Parasitology, Universidade Federal de Minas Gerais, Belo Horizonte, Minas Gerais, Brazil; 2 Instituto Nacional de Ciência e Tecnologia em Doenças Tropicais (INCT-DT), Brazil; 3 Laboratory of Cellular and Molecular Immunology, Centro de Pesquisas René Rachou, Fundação Oswaldo Cruz, Belo Horizonte, Minas Gerais, Brazil; 4 Clinical Hospital, Universidade Federal de Minas Gerais, Belo Horizonte, Minas Gerais, Brazil; 5 School of Nursing, Universidade Federal de Minas Gerais, Belo Horizonte, Minas Gerais, Brazil; Leiden University Medical Center, The Netherlands

## Abstract

Hookworm infection is considered one of the most important poverty-promoting neglected tropical diseases, infecting 576 to 740 million people worldwide, especially in the tropics and subtropics. These blood-feeding nematodes have a remarkable ability to downmodulate the host immune response, protecting themselves from elimination and minimizing severe host pathology. While several mechanisms may be involved in the immunomodulation by parasitic infection, experimental evidences have pointed toward the possible involvement of regulatory T cells (Tregs) in downregulating effector T-cell responses upon chronic infection. However, the role of Tregs cells in human hookworm infection is still poorly understood and has not been addressed yet. In the current study we observed an augmentation of circulating CD4^+^CD25^+^FOXP3^+^ regulatory T cells in hookworm-infected individuals compared with healthy non-infected donors. We have also demonstrated that infected individuals present higher levels of circulating Treg cells expressing CTLA-4, GITR, IL-10, TGF-β and IL-17. Moreover, we showed that hookworm crude antigen stimulation reduces the number of CD4^+^CD25^+^FOXP3^+^ T regulatory cells co-expressing IL-17 in infected individuals. Finally, PBMCs from infected individuals pulsed with excreted/secreted products or hookworm crude antigens presented an impaired cellular proliferation, which was partially augmented by the depletion of Treg cells. Our results suggest that Treg cells may play an important role in hookworm-induced immunosuppression, contributing to the longevity of hookworm survival in infected people.

## Author Summary

The hookworm infection is characterized by the long-term survival of the parasite and the concomitant modulation of the host immunity. Among several mechanisms that may account for the suppression of T cell response, we here described the presence and role of T regulatory cells (also known as Tregs) in the human hookworm infection. Tregs are a minor subpopulation of CD4^+^ T-cells, which also express specific cell markers that allow its further identification (CD25 and FOXP3). Our results showed that hookworm infection induce an augmentation of Tregs in the peripheral blood, followed by the higher levels of circulating Treg cells expressing several markers and cytokines associated with cell regulation (CTLA-4, GITR, IL-10, TGF-β and IL-17). We also demonstrated that *in vitro* depletion of Tregs partially enhanced the naturally impaired cellular proliferation of lymphocytes from infected individuals after antigenic stimulation. Our results suggest that Treg cells may play an important role in hookworm-induced immunosuppression, contributing to the longevity of hookworm survival in infected people.

## Introduction

Human hookworm infection is mainly caused by the blood-feeding nematodes *Ancylostoma duodenale* and *Necator americanus*, which infects 576 to 740 million people worldwide, especially in the tropics and subtropics [1], [2], [3]. Hookworm is considered one of the thirteen poverty-promoting *neglected tropical diseases*, and the second most important parasitic infection of humans [4]. This infection takes a particularly devastating toll on the most vulnerable of the world's population, including children, productive men, and women of childbearing age [5], [6], [7]. Persistent blood and serum protein loss attributable to chronic hookworm infection are associated with anemia, malnutrition, and growth/cognitive retardation, resulting in the annually loss of tens of millions of disability adjusted life-years (DALYs) [8]. Despite its overall impact over the global public health system, the immunomodulatory mechanisms associated with hookworm survival on host's intestine in face of an immunologically hostile environment are not yet fully understood.

Human hookworm infection is a longstanding, chronic infection with complex life cycle stages and host-parasite interactions. Although this parasitic infection may seem unnoticed by the immune system, an intense T helper (Th) 2 phenotype immune response is mounted by the host against this helminthic infection [9]. In recent years, evidence has accumulated that the immune response to hookworms may not be a simple polarized and putatively protective Th2 response, but rather a mixture of Th1/Th2 responses, presenting significant levels of interferon gamma and IL-12 production [10]. In fact, hookworms have a remarkable ability to downmodulate the host immune response, protecting themselves from elimination and minimizing severe host pathology. The parasite may promote its survival by excreting/secreting a panel of molecular immunosuppressive agents and, possibly, by stimulating the appearance of regulatory T-cell populations. Among the most striking aspects already described of this downregulation is the ablation of parasite specific T cell proliferative responses (“hyporesponsiveness”) [11]. Indeed, hookworm infection in animal models has been classically associated with impaired lymphocyte proliferation, functional defects in antigen presentation/processing and increased secretion of nitric oxide [12]. Recently, Fujiwara *et al.* have also shown that human dendritic cells differentiation and maturation may also be downmodulated by these worms, contributing to the T cell hyporesponsive state observed in individuals chronically infected with *N. americanus* [13].

The discovery of regulatory T lymphocytes (Treg) that are actively involved in maintaining immune tolerance has recently led to new insights into mechanisms of tolerance breakdown and/or immunoregulation in human diseases, including those resulting from allergic, autoimmune, or infectious causes [14]. These cells have been shown to suppress cellular immune responses through direct contact with immune effector cells and by the production of regulatory cytokines, including TGF-β and IL-10 [15], [16]. In fact, Geiger et al. have shown that hookworm infection is accompanied by elevated levels hookworm antigen-specific IL-10 production dependent on parasite stage, as well as significantly higher levels of CD4^+^/CD25^+^ T-cells [11]. While studies in experimental models have provided evidence for increased FOXP3^+^ (forkhead box P3 transcription factor) Treg function during different helminth infections [17], [18], [19], the role of Tregs cells in human hookworm infection is still poorly understood and has not been addressed.

To investigate Treg activity in human hookworm infection, we have evaluated Treg frequencies, function and immune responses to hookworm antigens in *N. americanus*-infected individuals from a rural area of Minas Gerais state (Brazil). In the present study we described an augmentation of circulating CD4^+^CD25^+^FOXP3^+^ regulatory T cells in hookworm-infected individuals compared with healthy non-infected donors. We have also demonstrated by flow cytometry that infected individuals present higher levels of circulating Treg cells expressing CTLA-4 (cytotoxic T lymphocyte antigen 4), GITR (glucocorticoid-induced tumor necrosis factor receptor), IL-10, TGF-β and IL-17. Additionally, we showed that hookworm crude antigen stimulation reduces the number of CD4^+^CD25^+^FOXP3^+^ T regulatory cells co-expressing IL-17 in infected individuals. Furthermore, PBMCs from infected individuals pulsed with excreted/secreted products or hookworm crude antigens presented an impaired cellular proliferation, which was augmented after the depletion of Treg cells. Taken together, our results suggest that Treg cells may play an important role in hookworm-induced immunosuppression, contributing to the longevity of hookworm survival in infected people.

## Materials and Methods

### Study population

The study was conducted in areas endemic for *N. americanus* in the Northeast Minas Gerais State, Brazil. Ten volunteers between the ages of 18 and 76 were recruited over the course of two months (Table 1). These volunteers reside in areas of moderate *N. americanus* transmission and presented with low to moderate (up to 872 epg) intensity of *Necator* infection. Individuals were selected on the basis of not having any other helminth infection (mono-infected after analysis of 6 slides of Kato-Katz fecal thick-smear and Baermann-Moraes techniques). The presence of *Necator* infection was determined by formalin–ether sedimentation and, if positive, two more stool samples were analyzed by the Kato–Katz fecal thick-smear technique and parasite load was expressed as eggs per gram of feces (epg) [20]. Ten hookworm-naive individuals were enrolled as healthy non-infected individuals from Belo Horizonte, Minas Gerais State, Brazil, where no transmission occurs. None of these individuals had a history of *Necator* infection and all presented with egg-negative stool (6 slides of Kato-Katz fecal thick-smear and Baermann-Moraes techniques) and no specific antibodies to *Necator* crude antigen extracts. The geographic areas included in this study are not endemic for tissue-dwelling helminth infections. Furthermore, the nutritional status of non-infected volunteers (controls) was similar to those presented by hookworm-infected individuals as determined by anthropometric measurements. The nutritional status of adults was determined using the absolute body mass index and classified as eutrophic (18.5–24.9 kg/m^2^), underweight (<18.5 kg/m^2^) or overweight (≥25 kg/m^2^) [21].

**Table 1:** Description of the study population by age, intensity of infection and hematological parameters (Mean and range).

	Individuals	
	*Necator*-infected(n = 10)	Non-infected(n = 10)
Age mean, years	35.35 (18–76)	36.25 (30–55)
Intensity of infection*	109.66 (4–872)	0
Hemoglobin(g/dL)	14.43 (11.8–16.4)	14.52 (14–16.5)
Whole blood count (cell/mm^3^)	6,821 (4,200–12,100)	7,600 (5,300–10,700)
Eosinophil (cell/mm^3^)	395.63 (59.4–1,711.3)**	180.7 (108–303)
Eosinophil (%)	5.43% (0.9–15.7)**	2.4% (2.0–4.0)
Lymphocytes (cell/mm^3^)	2,248.6 (1,055.6–3,368.9)**	3,116.3 (2,300.0–4,462.0)
Lymphocytes (%)	31.23% (17.1–57.1)**	36% (25.0–46.0)

*number of eggs per gram of feces.

**Statistically different from control group (p<0.05).

Approximately 24 mL of blood was collected in heparinized tubes for separation of peripheral blood mononuclear cells (PBMC) and 4 mL of blood in EDTA tubes for the immunological assays described below.

### Ethics statement

The study was approved by the Ethical Committee on Research of Universidade Federal de Minas Gerais (COEP) (Protocol #ETIC0449.0.203.000-09). Written consent was obtained from all individuals prior to enrollment in this study. *Ancylostoma ceylanicum* adult worms were obtained from purpose-bred hamsters maintained at the Universidade Federal de Minas Gerais according to a protocol approved by the Committee for Animal Experimentation of Universidade Federal de Minas Gerais (Protocol# 66/08). All animals procedures were performed under the guidelines from COBEA (Brazilian College of Animal Experimentation) and strictly followed the Brazilian law for “Procedures for Scientific Use of Animals” (11.794/2008).

### Hookworm antigen preparation

For preparation of excreted-secreted (ES) antigens, worms were removed from the intestines of euthanized hamsters, washed several times in phosphate-buffered saline (PBS), and then cultured overnight in RPMI 1640 containing 100 U/ml penicillin G sodium, 100 µg/ml streptomycin sulfate, and 0.25 µg/ml amphotericin B (all reagents from Sigma-Aldrich, St. Louis, MO) at 37°C with 5% CO_2_ in a humidified incubator. The ES products were concentrated using microconcentration filter units with a 10-kDa-cutoff membrane (Millipore, Bedford, MA). Adult worm crude extract was prepared by direct maceration of parasites using a tissue grinder and further rupture using a cell disruptor (Sonifier Cell Disruptor, Branson Sonic Power Co., Danbury, CT, USA) in PBS for 1 min at 40 Watts, in an ice bath. The procedure was repeated five times, with 1 min intervals between disruptions.

All of the antigen preparations used in cell cultures were passed through a 0.22 µm low-protein binding syringe filter (Millipore, USA), and the resulting protein concentration was determined using a BCA protein assay kit (Pierce, USA). Hookworm antigen preparations were tested negative for endotoxin content by the Limulus lysate assay (sensitivity of 0.06 U/ml; Cambrex, USA), and stored in aliquots at −80°C.

### *Ex vivo* flow cytometric analysis of peripheral blood

Whole blood was collected in Vacutainer tubes containing EDTA (Becton Dickinson, USA) and 100 µL samples were mixed in tubes with 2 µL of undiluted monoclonal antibodies PerCP anti-human CD4 (clone BNI3), FITC anti-human CD25 (clone M-A251) and APC anti-human FOXP3 (clone 236A) (all from BD Pharmingen, USA). After adding the antibodies, the cells were incubated in the dark for 30 minutes at room temperature. Following incubation, erythrocytes were lysed using 2 mL of FACS Lysing Solution (BD Biosciences, USA) and washed twice with 2 mL of phosphate-buffered saline containing 0.01% sodium azide and 0.5% bovine serum albumin (SIGMA, USA). Intracellular staining was performed after cell fixation in formaldehyde (4%) and permeabilization with saponin buffer (0.5%) (Sigma, USA) for 15 minutes. Cells were washed twice with 2 mL of phosphate-buffered saline containing 0.01% sodium azide and 0.5% bovine serum albumin (SIGMA, USA) and incubated for 30 minutes with 2 µL of undiluted monoclonal antibodies PE anti-human IL-10 (clone JES3-9D7), PE anti-human TGF-β (clone TB21), PE anti-human IL-17 (clone 64CAP17), PE anti-human CTLA-4 (clone BNI3) and PE anti-human GITR (clone eBioAITR) (all from BD Pharmingen, USA).

After incubation and washing with PBS with 0.01% sodium azide and 0.5% bovine serum albumin, the cells were the fixed in 200 µL of fixative solution (10 g/L paraformaldehyde, 1% sodium-cacodylate, 6.65 g/L sodium chloride). Phenotypic analyses were performed by flow cytometry with a FACScalibur flow cytometer (BD Biosciences, USA). Data were collected on 30,000 events (gated by forward and side scatter) and analyzed using CellQuest® software (BD Biosciences, USA).

### Phenotypic analysis of whole blood cultures stimulated with hookworm antigens

Whole blood was stimulated *in vitro* with ES and crude antigens (5 µg/well) in RPMI 1640 media supplemented with 1.6% L-glutamine (Sigma, USA), 3% antibiotic-antimycotic (Invitrogen, USA), 5% of heat inactivated AB^+^ human serum (Sigma, USA), for 24 hours at 37°C with 5% CO_2_. Unstimulated cultures were used as negative controls. During the last 4 hours of culture, Brefeldin A (Sigma, USA) (10 µg/mL) was added to the cultures.

Phenotypic analyses were performed by flow cytometry after staining using the same antibody panel described for *ex vivo* immunophenotyping assays. Data were collected on 30,000 events (gated by forward and side scatter) and analyzed using CellQuest® software (BD Biosciences, USA).

### Isolation of PBMC and purification of CD4^+^CD25^+^ T cell subpopulation

Peripheral blood mononuclear cells (PBMC) were isolated by Ficoll-Hypaque (GE Healthcare, USA) using density gradient centrifugation (Sigma, USA). Cells were then washed and resuspended at 5×10^6^ cells/mL in RPMI 1640 medium (Invitrogen, USA), supplemented with 5% heat-inactivated human AB serum (Sigma, USA), 2 mM of L-glutamine (Sigma, USA), 50 U/mL of penicillin, and 50 g/mL of streptomycin (Invitrogen, USA).

CD4^+^CD25^+^ T cells were purified from PBMCs using the CD4^+^CD25^+^ regulatory T cell isolation kit and a QuadroMACS cell separator (both from Miltenyi Biotec, USA), according to the protocol provided by the manufacturer. In brief, cells were suspended in PBS supplemented with 2 mM EDTA and 0.5% BSA (Sigma, USA) at a density of 10^7^ cells in 90 µL of buffer and 10 µl of biotin-Ab mixture. Cells were incubated at 4°C for 10 minutes. Then, 20 µL of anti-biotin microbeads was added and incubated for 15 min at 4°C. CD4^+^ cells were eluted and resuspended at a density of 10^7^ cells in 90 µL of buffer and 10 µL of CD25 microbeads. The cells were then incubated at 4°C for 15 min and further separated by a magnetic field. CD4^+^CD25^+^ T cell fraction retained in the column was eluted by removing the column from the magnetic field and flushing out the cells with 1 mL elution buffer (PBS with 2 mM EDTA and 0.5% BSA). CD4^+^CD25^+^ T cells were washed and used immediately. The purity of CD4^+^CD25^+^ T cells after purification reached up to 95% (data not shown) (Supplementary Figure S1). CD4^+^CD25^−/low^ T cells and CD4^−^ cells together were considered as Treg-depleted PBMCs (dPBMCs) and used in functional assays.

### Functional analysis of the effect of CD4^+^CD25^+^ T cells from hookworm infected individuals on *in vitro* cellular proliferation

Stimulation assays were performed in duplicates and mitogen and antigens were added at previously determined concentrations known to result in optimal proliferation [22]. The mitogen phytoemagglutinin-l (PHA–L) (Sigma, USA) was used for polyclonal stimulation of peripheral blood mononuclear cells (PBMCs). Crude and excreted-secreted (ES) antigens from adult *Ancylostoma ceylanicum* were employed for hookworm specific cellular stimulation at a final concentration of 5 µg/well.

For the analysis of the effect of CD4^+^CD25^+^ T cells on cellular proliferation, two different experiments were performed. Firstly, PBMCs (10^6^ cells/mL in PBS/1% BSA) were co-labeled with 0.4 mM CFDA-SE (carboxyfluorescein diacetate succinimidyl ester, Vybrant™ CFDA-SE Cell Tracer Kit, Molecular Probes, USA) and PE-conjugated anti-human CD8 (clone UCTH-4) or PE-Cy5 anti-human CD4 (clone RPA-T4) (BD Pharmingen, USA) for 10 minutes at room temperature. The previously purified CD4^+^CD25^+^ T cells were incubated at ratio of 1∶10 with autologous CFDA-SE-labeled PBMCs pulsed with hookworm antigens, for 96 hours at 37°C with 5% CO_2_ atmosphere.

In a second experiment, Treg-depleted PBMCs were co-labeled with CFDA-SE/CD4 or CD8 and pulsed with hookworm antigens, as previously described. The cell proliferative response of both experiments was assessed using a FACScan® cytometer (Becton Dickinson, USA) and CellQuest® software (BD Biosciences, USA). Analysis of CFDA-SE proliferation was performed as previously described [23].

### Determination of cytokine production by sandwich ELISA

The cytokines IL-2, IFN-γ, IL-10, and IL-5 were detected and quantified in cell supernatants by commercially available sandwich ELISA kits (R&D Systems, USA). Assays were performed according to the manufacturer's instructions. Biotin-labeled detection antibodies were used, revealed with streptavidin-HRP (Amersham Biosciences, USA) and OPD substrate system (Sigma). The colorimetric reaction was read in an automated ELISA microplate reader at 492 nm. Calculations of chemokine/cytokine concentrations from mean optical density values were interpolated from the standard curve using 5-parameter curve fitting software (SOFTmax® Pro 5.3, Molecular Devices, USA). Results were achieved in pg/mL and the detection limits were as follows: 15.6 pg/mL for IL-2; 3.9 pg/mL for IFN-γ; 23.4 pg/mL for IL-10 and IL-5. Samples with values above the top of the standard curve were retested at 1/10 or 1/100 dilutions in RPMI 1640, and the chemokine/cytokine levels were recalculated.

### Statistical analysis

The one-sample Kolmogorov-Smirnoff test was used to determine whether variability followed a normal distribution pattern. The Mann-Whitney U test was used to determine the differences (p value<0.05) of non-parametric variables between *Necator*-infected individuals and non-infected individuals. The maximum residual test (Grubb's test) was used to detect possible outliers. All statistics were carried out using Prism 5.0 for Windows (GraphPad Software Inc., USA).

## Results

### Regulatory T cell (CD4^+^CD25^+^FOXP3^+^) frequency is increased in *N. americanus* infected donors

**Figure 1:**
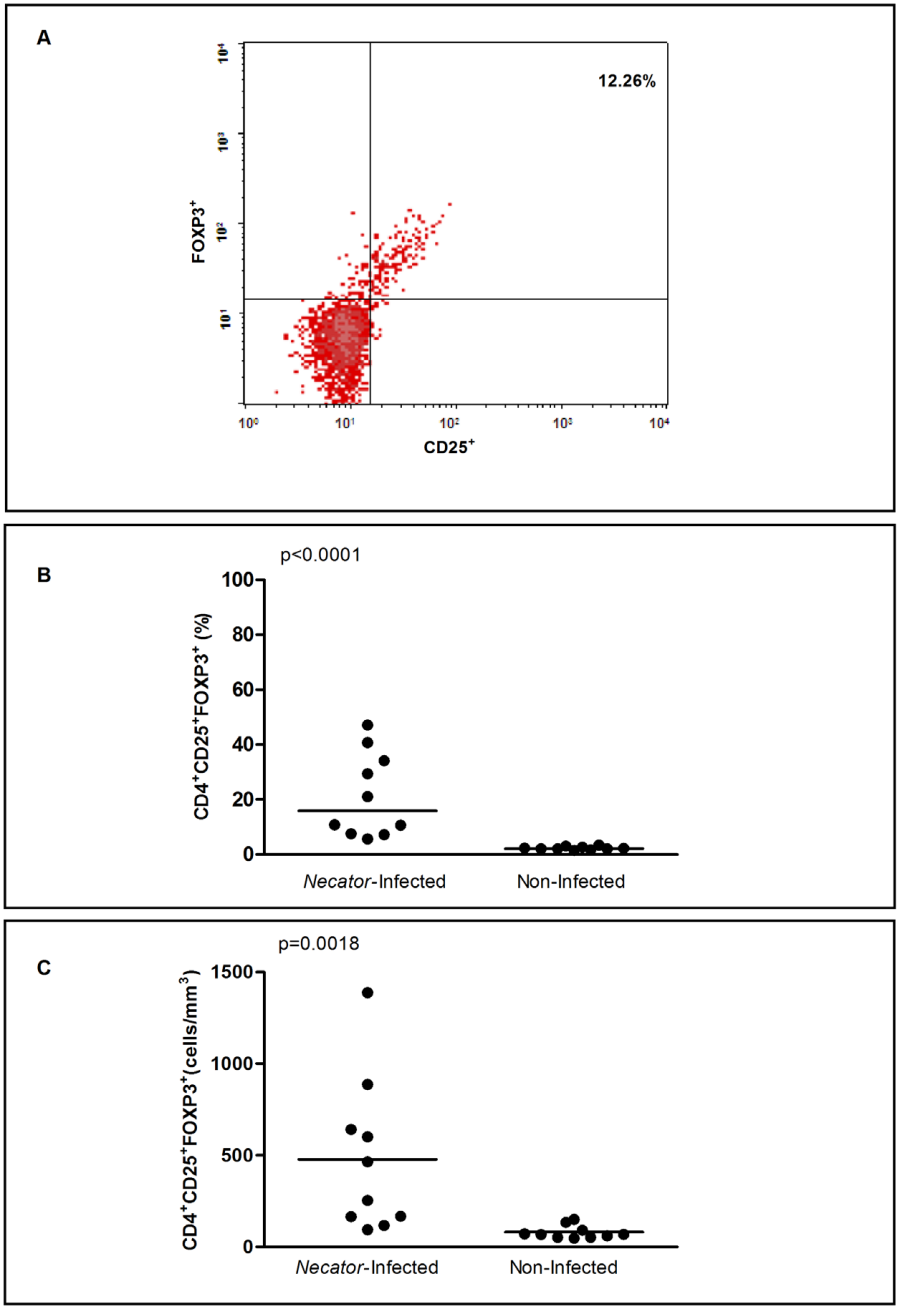
Flow cytometric analysis of regulatory T cells. (A) CD25 and FOXP3 expression in gated CD3^+^CD4^+^ lymphocytes. Dot plot shows a representative data of 20 donors examined. (B) Frequency and (C) absolute numbers of circulating CD4^+^CD25^+^FOXP3^+^ regulatory T cells in *Necator*-infected and non-infected donors (n = 10 for both groups). Frequency (%) and absolute numbers (cells/mm^3^) are indicated on Y-axis and lines represent mean. Statistical differences were detected using Mann-Whitney U test and are indicated on the graph with significant P values.

Regulatory T cells were identified by flow cytometry as CD4^+^ T cells expressing both CD25 and FOXP3 marker (Figure 1A) and are reported as frequency (Figure 1B) and absolute numbers of cells per mm^3^ (Figure 1C). Analysis of PBMCs from *Necator-*infected individuals showed a significant increase in frequency (p<0.0001, Figure 1C) and absolute numbers (p = 0.0018, Figure 1B) of circulating CD4^+^CD25^+^FOXP3^+^ T cells (21.4±15.4%, 477.4±131.8 cells/mm^3^) when compared with non-infected naive individuals (2.3±0.6%, 78.9±11.2 cells/mm^3^).

### Specific peripheral blood subpopulations of CD4^+^CD25^+^FOXP3^+^ T cells are elevated in *N. americanus*-infected individuals

**Figure 2:**
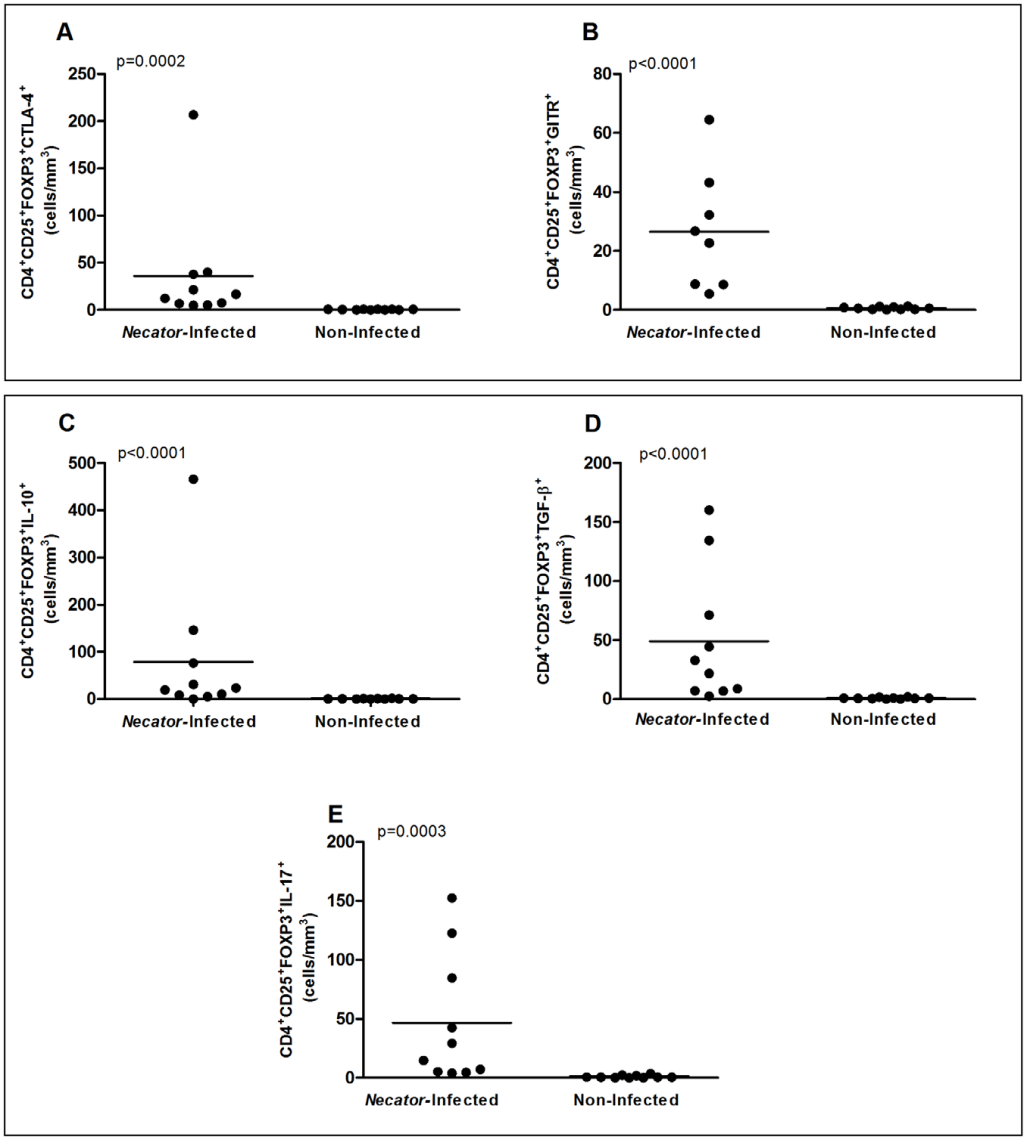
Flow cytometric analysis of surface markers (CTLA-4 and GITR) and cytokines (IL-10, TGF-β, and IL-17) in CD4^+^CD25^+^FOXP3^+^ regulatory T cells in *Necator*-infected and non-infected donors (n = 10 for both groups). Results were expressed as absolute numbers of cells expressing (A) CTLA-4, (B) GITR, (C) IL-10, (D) TGF-β, and (E) IL-17. Absolute numbers (cells/mm^3^) are indicated on Y-axis and lines represent median. Statistical differences were detected using Mann-Whitney U test and are indicated on the graphs with significant P values.

Once observed the elevated number of Treg cells in the peripheral blood of hookworm infected donors, we further characterized this cell population by evaluating the expression of molecules and cytokines associated with cell modulation. Surface expression of the GITR molecule and intracellular expression of CTLA-4, IL-10, TGF-β and IL-17 cytokines, were assessed by flow cytometry. Infection of *N. americanus* significantly increased the proportion of cells expressing CTLA-4 (p = 0.0002) and GITR (p<0.0001). Flow cytometric analysis also showed a significant augmentation of CD4^+^CD25^+^FOXP3^+^ cells producing IL-10 (p<0.0001), TGF-β (p<0.0001) and IL-17 (p = 0.0003) in hookworm infected individuals (Figure 2). Similar results were found when frequency of cells were analyzed (Supplementary Figure S2).

### Expression of surface markers and cytokines on CD4^+^CD25^+^FOXP3^+^ is similar between infected and control individuals

The expression of analyzed surface and intracellular markers was determined by median intensity of fluorescence in order to obtain the absolute expression level per cell basis. Conversely to the increase in the absolute numbers of Treg subpopulations, no differences in the expression of IL-10, TGF-β, IL-17, CTLA-4 and GITR, between infected and non-infected individuals were observed (data not shown).

### Stimulation of whole blood cultures with hookworm crude antigen reduces the percentage of CD4^+^CD25^+^FOXP3^+^IL-17^+^ cells

**Figure 3:**
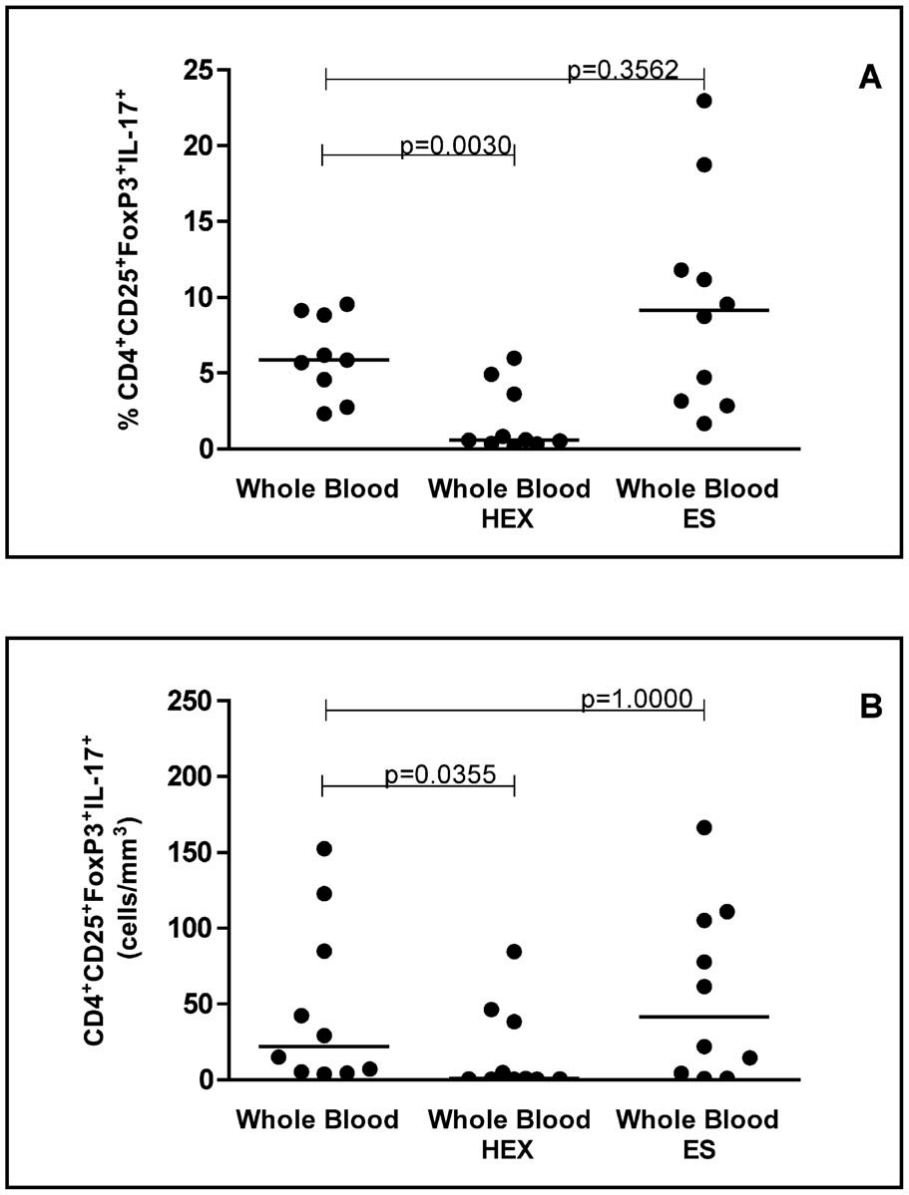
Effect of direct stimulation of whole blood with hookworm antigens in the CD4^+^CD25^+^FOXP3^+^ regulatory T cells producing IL-17. (A) Frequency (%) and (B) absolute numbers of cells (cells/mm^3^) of CD4^+^CD25^+^FOXP3^+^IL-17^+^ T cells are indicated on Y-axis. Hookworm adult crude extract (HEX) and excretory-secretory (ES) products were used in the whole blood cultures of *Necator*-infected donors (n = 10). Statistical differences were detected using Mann-Whitney U test and are indicated on the graph with significant P values.

In order to determine the possible effect of hookworm antigens on the expression of cell surface markers (CTLA-4 and GITR) and cytokines (IL-17, TGF-β, and IL-10) by CD4^+^CD25^+^FOXP3^+^ regulatory T cells in *N. americanus*-infected donors, whole blood cultures were stimulated with either hookworm crude antigen or ES products. When crude antigen was added to the *in vitro* cultures, the percentage and the absolute counts of CD4^+^CD25^+^FOXP3^+^ regulatory T cells co-expressing IL-17 was significantly reduced (p<0.0030) (Figure 3). Although there was a tendency in increase on the expression of IL-17 in ES stimulated blood it was not statistically significant (p = 0.3562) (Figure 3). No differences in the proportion of cells expressing CTLA-4, GITR, TGF-β, IL-10, (Supplementary Figure S3) and median intensity of fluorescence were seen after hookworm antigen stimulation (p>0.05 for all).

### CD4^+^CD25^+^ Treg cells modulates antigen-specific PBMC proliferation in *N. americanus*-infected individuals

**Figure 4:**
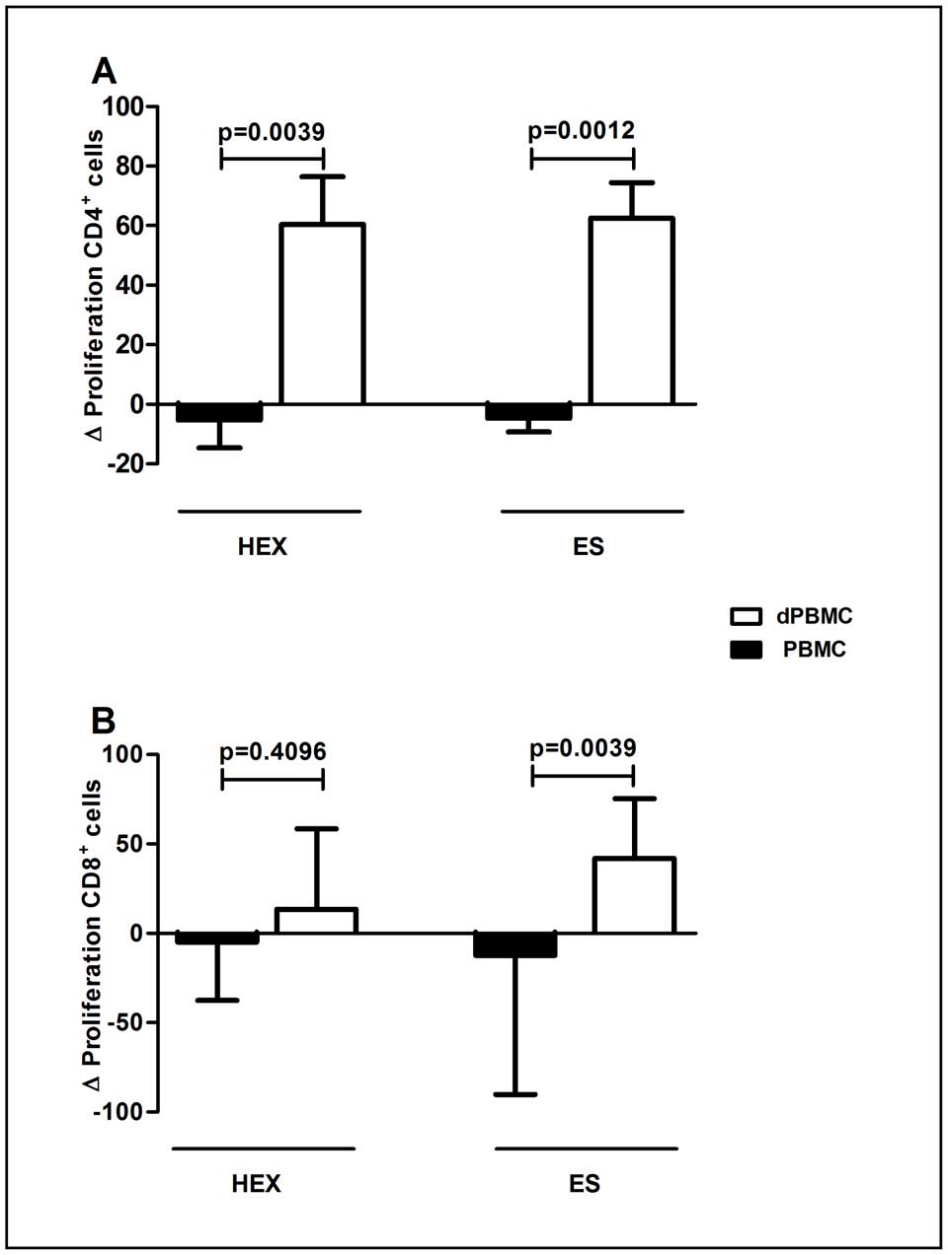
Depletion of CD4^+^CD25^+^ lymphocytes augments the *in vitro* cellular proliferation of CD4^+^ and CD8^+^ lymphocytes after parasite antigen stimulation. ΔCFDA-SE Proliferation of (A) CD4^+^ or (B) CD8^+^ in PBMCs and Treg-depleted PBMCs (dPBMCs) from hookworm infected donors (n = 10) after stimulation with hookworm adult crude extract (HEX) and excretory-secretory (ES) products. ΔCFDA-SE Proliferation was calculated by proliferative response observed in stimulated PBMCs/dPBMCs (indicated by positivity for CFDA-SE) subtracted from basal proliferative response of non-stimulated cells (PBMCs or dPBMCs only). Statistical differences were detected using Mann-Whitney U test and are indicated on the graph with significant P values.

Chronic human *N. americanus* infection is classically associated with a profound ablation of cell proliferation. In order to determine the possible effect of Treg cells on the immune response during hookworm infection, functional assays were designed to evaluate whether CD4^+^CD25^+^FOXP3^+^ Treg cells could modulate the *in vitro* cellular proliferation of CD4^+^ and CD8^+^ lymphocytes after parasite antigen stimulation. Hookworm antigen-stimulated PBMCs from infected individuals showed a naturally impaired proliferative response, which was not further suppressed by co-incubation with Tregs (data not shown). However, *in vitro* cultures, where Tregs cells were depleted (dPBMC), showed a significant increase on the CD4^+^ cell proliferative response induced by crude (p = 0.0039) and ES (p = 0.0012) hookworm antigenic stimulation (Figure 4A). Interestingly, depletion of Treg cells significantly augmented the proliferation of CD8^+^ PBMCs of infected donors in response to hookworm ES products (p = 0.0039), but not to crude antigen (Figure 4B). The depletion of Tregs elicited the increase of IL-2 and lower levels of IL-10 in supernatants of dPBMCs with or without antigenic stimulation although statistical significance was not achieved (Supplementary Figure S4). No differences were also observed for IFN-γ and IL-5 (Supplementary Figure S4) after CD4^+^CD25^+^ depletion. These results suggest that T regulatory cells do have the capacity to modulate the *in vitro* proliferative response in hookworm-infected patients. Additional experiments using PBMCs from control individuals demonstrated the absence of cell proliferative response after antigenic stimulation, which remains unaltered by the add-back or depletion of Tregs (Supplementary Figure S5). No differences on cell proliferative response of PBMCs from both infected and control individuals were observed in cultures after stimulation with the mitogen PHA–L.

## Discussion

CD4^+^CD25^+^FOXP3^+^ regulatory T cells (Tregs) constitute a minor subpopulation of CD4^+^ T-cells, which play an important role in controlling the extent of the immune-mediated pathology and maintaining immunological self-tolerance and immune homeostasis [24], [25]. These cells suppress the activation and proliferation of CD4^+^ and CD8^+^ T cells by direct contact of with effector T cells or secretion of immunoregulatory cytokines, such as IL-10 and TGF-β [16], [26]. Moreover, the balance of Treg cell–dependent immunomodulation may lead to enhanced pathogen survival and, in some cases, their long-term persistence [27]. In fact, one of the hallmarks of chronic helminth infections is induction of T-cell hyporesponsiveness and bystander suppression [28]. While the mechanisms involved in the immunomodulation by parasitic infection may be multiple, some experimental evidences have pointed toward the possible involvement of natural and inducible Treg in downregulating effector T-cell responses upon chronic infection. Over the past four decades [29], [30], several studies have attempted to describe the role of regulatory T cells in parasitic diseases (reviewed in [16], [27]), including leishmaniasis, schistosomiasis, malaria and lymphatic filariasis. However, a limited number of studies have focused on the currently known Treg dynamics and functional capacity in human helminth infections.

Evidence for Treg activity in human chronic helminth infections has been firstly provided by T cell clones generated from onchocerciasis patients [31] and recently described for geohelminth infection in humans [32]. Several studies in animal models of filariasis [33] and schistosomiasis [34], [35], demonstrated that Treg phenotype populations develop following infection, whilst in infection with the murine gastrointestinal nematode *Heligmosomoides polygyrus* [36], functional regulation by CD4^+^CD25^+^ T cells suppresses the bystander response to an allergic provocation. In the present study we describe the role of CD4^+^CD25^+^FOXP3^+^ T cells in human *N. americanus* infection. To explore cellular immune mechanisms underlying classic hookworm-induced T cell hyporesponsive state, we have analyzed Treg frequencies in peripheral blood and performed *in vitro* Treg depletion and add-back experiments with PBMC isolated from *N. americanus*-infected individuals from a rural area of Minas Gerais State, Brazil.

We initially showed that hookworm-infected individuals present a significant increase of circulating Treg cells in peripheral blood compared to non-infected healthy volunteers, as previously demonstrated in other nematode infections [18], [19], [37], [38], [39]. Of note, while the expansion of CD4^+^CD25^+^FOXP3^+^cells is observed in infected individuals, no differences were observed in the expression of all markers associated with cell suppression, including FOXP3. Such increase in the absolute number of circulating FOXP3^+^ Treg cells might be driven as a direct consequence of the infection. In fact, the expression of FOXP3^+^ in naïve T cells can be elicited by excretory-secretory products of nematodes, resulting in induced *de novo* FOXP3^+^ expression and active suppressor cells [17]. Interestingly, hookworm-infected individuals also presented with significant lower levels of circulating lymphocytes, which may be partially explained by the inhibition of effector T cells emergence during the inductive phase of the immune response in the secondary lymphoid tissues by IL-10-independent mechanisms [40]. It is possible that increased Treg activity may trigger modulation of host immune response and consequently facilitate hookworm prolonged survival. On the other hand, increased Treg responses might also account for limitation of exacerbated infection-induced tissue pathology, which would be ultimately beneficial to the host. However, it would not limit the blood loss or anemia induced by the infection.

A variety of potential mediators of Treg activity that could contribute to the suppression of the host's immune response have been identified, including GITR [41], [42], CTLA-4 [43], FOXP3 [44], [45], and the anti-inflammatory cytokines IL-10 and TGF-β [46], [47], [48]. In the current study, a significant increase of circulating CD4^+^CD25^+^FOXP3^+^ lymphocytes, co-expressing GITR, CTLA-4, IL-10, TGF-β or IL-17, was demonstrated in *N. americanus*-infected donors, compared to non-exposed volunteers. Nevertheless, a higher expression of these markers on per cell basis has not been observed in hookworm-infected donors in relation to healthy individuals.

Both GITR and CTLA-4 molecules are constitutively expressed on cell surface of natural Tregs [27] and are regulated by FOXP3 expression [49], [50]. Initial studies related to the effects of GITR signaling on Treg cells indicated that interaction of this receptor with anti-GITR antibody or GITR ligand (GITRL) lead to an apparent abrogation of suppressive activity of Tregs [41], [42], [51]. Indeed, treatment of *Trichuris muris* infected mice with anti-GITR resulted in an earlier worm expulsion [19]. Although not essential for the T cell suppressor activity [50], the engagement of GITR promotes proliferation of Tregs [50], [52] and potential enhancement of their suppressive function [51]. The significant increase of circulating CD4^+^CD25^+^FOXP3^+^GITR^+^ and CD4^+^CD25^+^FOXP3^+^CTLA-4^+^ in *Necator*-infected donors might partially reflect the concomitant augmentation of Tregs. Similarly, mice experimentally infected *H. polygyrus* or *Litomosoides sigmodontis* also presented a prominent increase of GITR and CTLA-4 expression [39], [53]. The inhibitory receptor CTLA-4 presents partial homology to CD28 molecule and interacts to the same ligands, CD80 and CD86, with a much higher affinity [54]. The suppressive effect of CTLA-4 is associated with the reduced IL-2 production and IL-2 receptor expression, and by arresting T cells at the G1 phase of the cell cycle [55], [56]. Moreover, CTLA-4 expressing Treg cells induce the expression of the enzyme indoleamine 2,3-dioxygenase (IDO) by antigen-presenting cells which degrades tryptophan, and the lack of this essential amino acid inhibits T cell activation and promotes T cell apoptosis [57]. In helminth infections, such as lymphatic filariasis, the expansion of CTLA-4^+^ T cell populations in was associated with suppressed T cell function [58]. The increased number of circulating GITR^+^ and CTLA-4^+^ Treg cells in hookworm-infected individuals suggests that these cells might play a suppressive role on host immune regulation.

Although it has long been recognized that IL-10-producing T cells could be generated *in vivo* during parasitic infection [53], it is only recently that the concept has emerged that specialized subsets of regulatory T cells contribute to this regulatory network [16]. In the current study, hookworm-infected individuals presented a significant augmentation of CD4^+^CD25^+^FOXP3^+^cells producing the anti-inflammatory cytokines IL-10 and TGF-β. In fact, a prominent rise in IL-10 secretion was demonstrated in ES antigen-stimulated PBMC cultures during primary experimental and natural human hookworm infection [22], [59]. It is well known that IL-10 and TGF-β are naturally produced by Tregs [27] and are required to induce FOXP3 expression [60]. Although it is not clear whether or how precisely hookworm infection influences the production these anti-inflammatory cytokines, lower levels of IL-10 were observed in supernatants of cultures after Treg depletion, which might support the possibility that that *N. americanus* induced-Tregs contribute as an important source of their production.

In this study, a significant increase of circulating CD4^+^CD25^+^FOXP3^+^cells producing the IL-17 was observed in infected donors, corroborating previous studies on other parasitic diseases [15], [17], [60]. Noteworthy, a recent study demonstrated that human hookworm products are able to influence the pro-inflammatory Th17 pathway, promoting a significant decrease in IL-17 production in the mouse infection model [61]. Moreover, it has been suggested that worm infection could block mucosal IL-23 and IL-17 secretion, leading to an important mechanism of control of inflammatory responses [62]. In fact, Ruyssers et al. also observed that helminth antigens could reduce the expression of IL-17 in both colon and mesenteric lymph node T cells [63]. Interestingly, a significant reduction in CD4^+^CD25^+^FOXP3^+^IL-17^+^ T cells after hookworm crude antigen stimulation was also demonstrated, suggesting the possible secretion of this cytokine or downmodulation of IL-17 expression after cell restimulation. Indeed, Elliott et al. showed that mouse colonization with the helminth *H. polygyrus* reduces IL-17A mRNA expression by mesenteric lymph node (MLN) cells and inhibits IL-17 production by cultured lamina propria mononuclear cells and MLN cells [64]. Moreover, the co-expression of FOXP3 and IL-17 may indicate the transient status of CD4^+^ lymphocytes from hookworm-infected individuals between Treg (FOXP3) and Th17 (IL-17) profiles, where the development of either pathway of differentiation is driven by TGF-β and IL-6 [65]. The decrease of IL-17 after antigenic stimulation might imply the differentiation of these transient cells in truly effector Tregs. Nonetheless, our results suggest that hookworm products are able to induce an immunomodulated microenvironment at the site of infection. Although recent evidences demonstrate the local suppression of Th1 and Th17 inflammatory cytokines during hookworm infection [66], the role of IL-17 in hookworm-induced Tregs still remains to be addressed.

Based in our results, we demonstrated that hookworm infected individuals present a significant augmentation of activated Treg cells in the peripheral blood, observed by increased numbers of Treg subpopulations expressing cell surface molecules and mediators associated with suppression of immune responses. These cells might contribute to the suppressive effect of this parasitic infection, leading to reduction of antigen-specific proliferative responses previously demonstrated in human populations and animal models [13], [67]. Indeed, chronic human *N. americanus* infection has classically been associated with a profound ablation of cell proliferation, which may even extend to other infectious agents and mitogens (“bystander effect”) [22], [68]. In the present study, we have demonstrated by functional assays that both T CD4^+^ and CD8^+^ cell proliferative responses to either ES or hookworm crude antigen were increased after Treg depletion, followed by an increase of IL-2 secretion and lower levels of IL-10, although not statistically significant, further implying that these cells may exert a specific immunomodulatory effect during persistent hookworm infection. Recently, Cuéllar et al. showed that coincubation of mouse splenic T cells with dendritic cells pulsed with the hookworm antigen Ac-TMP-1 induced their differentiation into CD4^+^ and, particularly, CD8^+^CD25^+^FOXP3^+^ T cells that expressed IL-10 [69]. These cells were able to suppress proliferation of naive and activated CD4^+^ T cells by TGF-β-dependent (CD4^+^ suppressors) or independent (CD8^+^ suppressors) mechanisms. However, while we showed that the expansion and suppressive effects of Tregs are prominent during chronic hookworm infection, no changes in the number of T cells nor in the absolute counts of regulatory T cells were observed in the human primo-infection with *N. americanus* [70]. Nonetheless, the data in the present work suggest that hookworms exploit Treg cells to facilitate its own survival by dampening host immune response. In conclusion, hookworm-induced Treg activity may be able to control and divert selective proliferative and cytokine responses to numerous disorders, such as intestinal inflammation, airways inflammation/hyper-reactivity, diabetes, and multiple sclerosis. While it is known that ES products from nematodes may stimulate T reg cells [17], only few studies have demonstrate the immunomodulatory properties of hookworm-derived antigens [38], [71]. Considering the recent availability of transcriptomic data sets for hookworm species [72], [73], further studies are still required to identify specific antigens directly associated with host's immune suppression, leading to the understanding of mechanisms used by the parasite to skew the immune response in its favour and the possible discovery of several promising candidate vaccine antigens.

While our results shed light on the patent mechanisms of immunosuppression present in hookworm infection, it is important to mention that the sample size and age variation of our studied population might be considered as possible limiting factors of our study. Moreover, although effort was made to match the nutritional status of all participants (endemic and non-endemic areas), unfortunately perfect matching of control individuals and infected patients by age and sex was not always possible. The absence of negative individuals from endemic areas as controls was preferred once it is not possible to guarantee the absence of infection in these donors (due to the limited sensitivity of fecal exams or long pre-patency period). Moreover, it has been previously shown that the immunological status of helminth-infected patients remain unaltered after anthelmintic treatment for several months [71]. Nonetheless, this study was designed to minimize potential confounders, which could mask the immunological assessment of hookworm infection. Therefore, our data should be further validated by large immunoepidemiological surveys to be conducted in endemic areas.

## Supporting Information

Figure S1 **FACS analysis.** Representative FACS dot plots for 1 out 20 donors expressing the purity of CD4^+^CD25^+^ T cells after purification. (A) CD4^+^CD25^+^ cells were initially gated within lymphocyte population based on their FSC/SSC distribution. (B) Frequency of CD4^+^CD25^+^ cells (double positive population) after purification.
(TIF)

Figure S2 **Flow cytometric analysis of surface markers (CTLA-4 and GITR) and cytokines (IL-10, TGF-β, and IL-17) in CD4^+^CD25^+^FOXP3^+^ regulatory T cells in** ***Necator*** **-infected and non-infected donors (n = 10 for both groups).** Results were expressed as frequency (%) of gated cells expressing (A) CTLA-4, (B) GITR, (C) IL-10, (D) TGF-β, and (E) IL-17. Frequency is indicated on Y-axis and lines represent median. Statistical differences were detected using Mann-Whitney U test and are indicated on the graphs with significant P values.
(TIF)

Figure S3 **Effect of direct stimulation of whole blood with hookworm antigens in the CD4^+^CD25^+^FOXP3^+^ regulatory T cells producing CTLA-4, GITR, IL-10 and TGF-β.** Frequency (%) of (A) CD4^+^CD25^+^FOXP3^+^CTLA-4^+^, (B) CD4^+^CD25^+^FOXP3^+^GITR^+^, (C) CD4^+^CD25^+^FOXP3^+^IL-10^+^ and (D) CD4^+^CD25^+^FOXP3^+^TGF-β^+^ are indicated on Y-axis. Hookworm adult crude extract (HEX) and excretory-secretory (ES) products were used in the whole blood cultures of *Necator*-infected and non-infected donors (n = 10 for both groups).
(TIF)

Figure S4 **Cytokine concentrations (mean ± standard error) of IL-2 (A), IFN-γ (B), IL-10 (C) and IL-5 (D) in supernatants from cultures (n = 10) of PBMCs and CD4^+^CD25^+^-depleted PBMCs (dPBMCs) after stimulation with crude extract (HEX) and excretory-secretory (ES) products.** Results are expressed in pg/mL.
(TIF)

Figure S5 **ΔCFDA-SE Proliferation of (A) CD4^+^ or (B) CD8^+^ in PBMCs and Treg-depleted PBMCs (dPBMCs) from control egg-negative donors (n = 10) after stimulation with hookworm adult crude extract (HEX) and excretory-secretory (ES) products.** ΔCFDA-SE Proliferation was calculated by proliferative response observed in stimulated PBMCs/dPBMCs (indicated by positivity for CFDA-SE) subtracted from basal proliferative response of non-stimulated cells (PBMCs or dPBMCs only.
(TIF)
