# Supplementary figures and images for: Induction of CD4+CD25+FOXP3+ Regulatory T Cells during Human Hookworm Infection Modulates Antigen-Mediated Lymphocyte Proliferation
Source: PLoS Negl Trop Dis. 2011 Nov 8;5(11):e1383. doi: 10.1371/journal.pntd.0001383 (PMC3210756; doi:10.1371/journal.pntd.0001383)

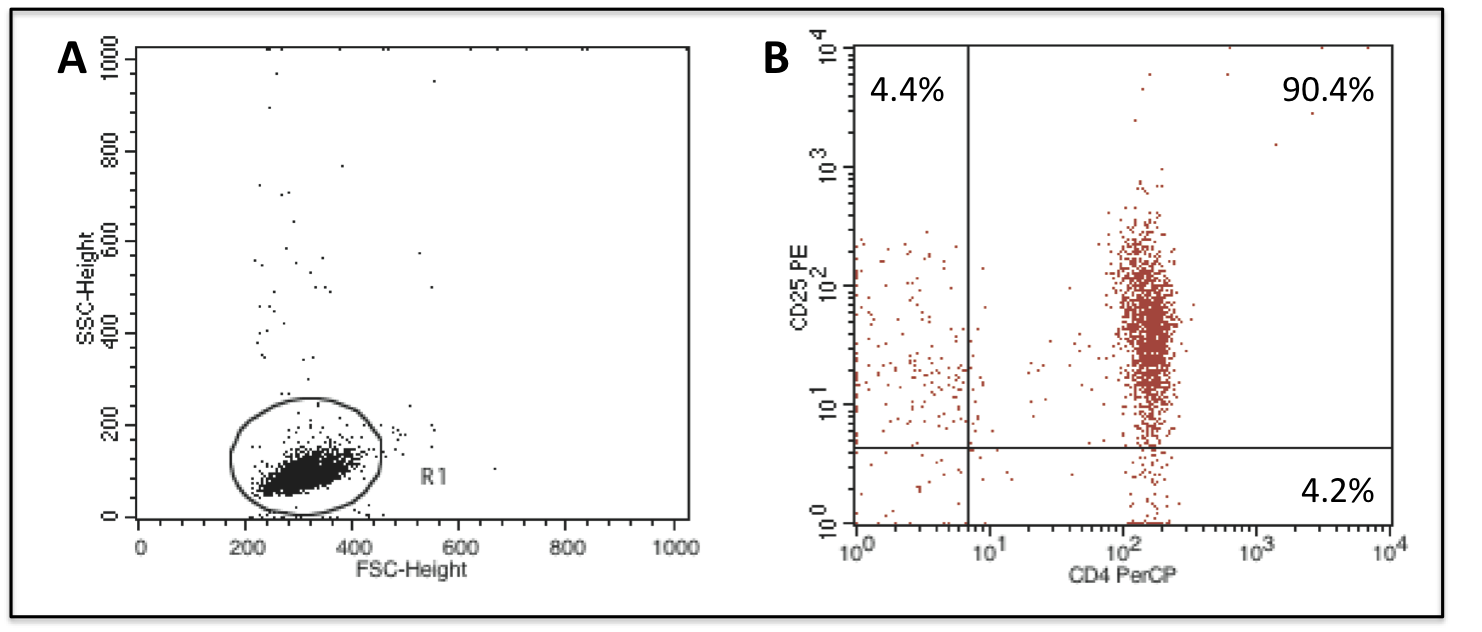

Supplement: Figure S1 — FACS analysis. Representative FACS dot plots for 1 out 20 donors expressing the purity of CD4+CD25+ T cells after purification. (A) CD4+CD25+ cells were initially gated within lymphocyte population based on their FSC/SSC distribution. (B) Frequency of CD4+CD25+ cells (double positive population) after purification. (TIF) [file pntd.0001383.s001.tif]

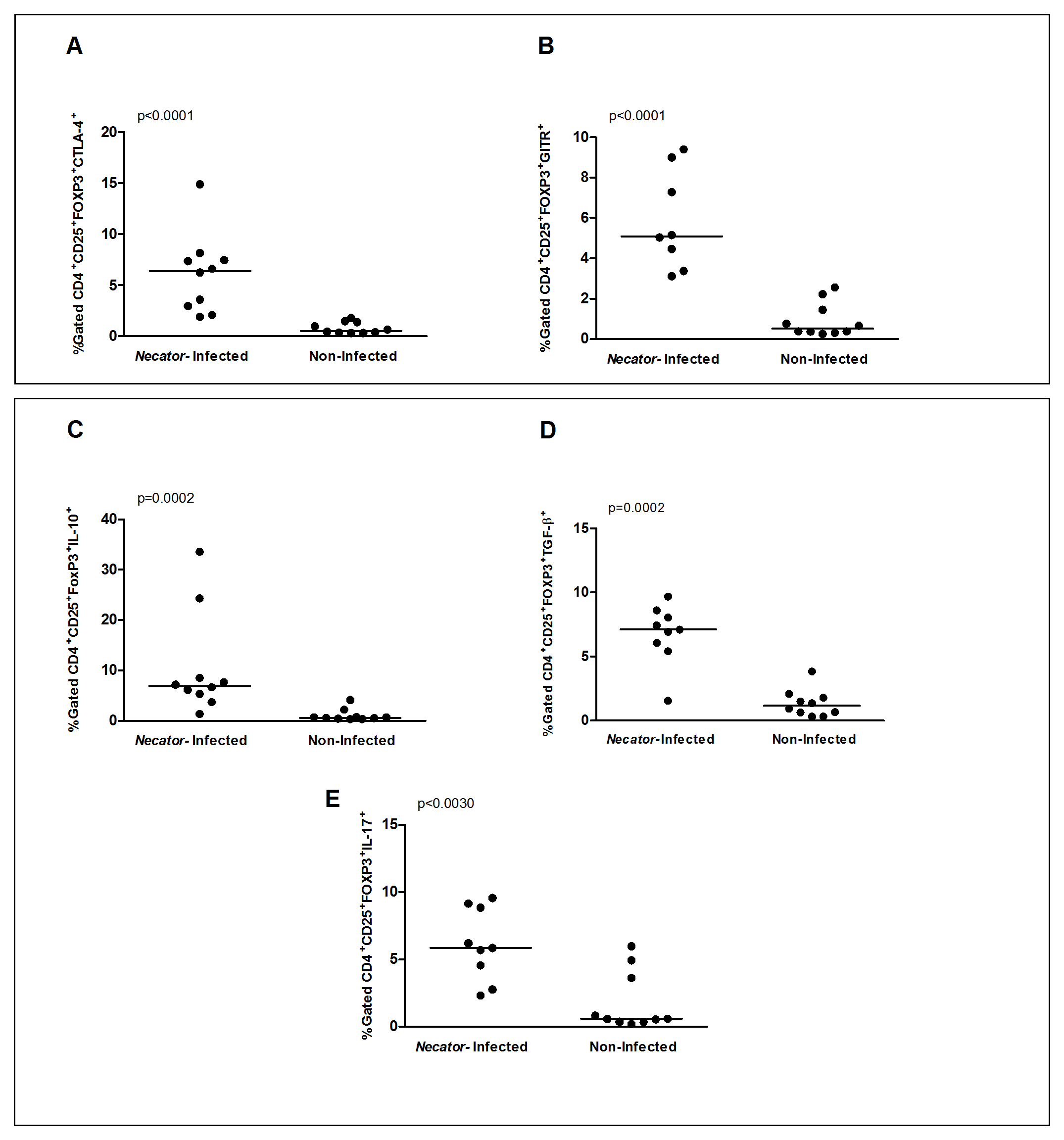

Supplement: Figure S2 — Flow cytometric analysis of surface markers (CTLA-4 and GITR) and cytokines (IL-10, TGF-β, and IL-17) in CD4+CD25+FOXP3+ regulatory T cells in Necator -infected and non-infected donors (n = 10 for both groups). Results were expressed as frequency (%) of gated cells expressing (A) CTLA-4, (B) GITR, (C) IL-10, (D) TGF-β, and (E) IL-17. Frequency is indicated on Y-axis and lines represent median. Statistical differences were detected using Mann-Whitney U test and are indicated on the graphs with significant P values. (TIF) [file pntd.0001383.s002.tif]

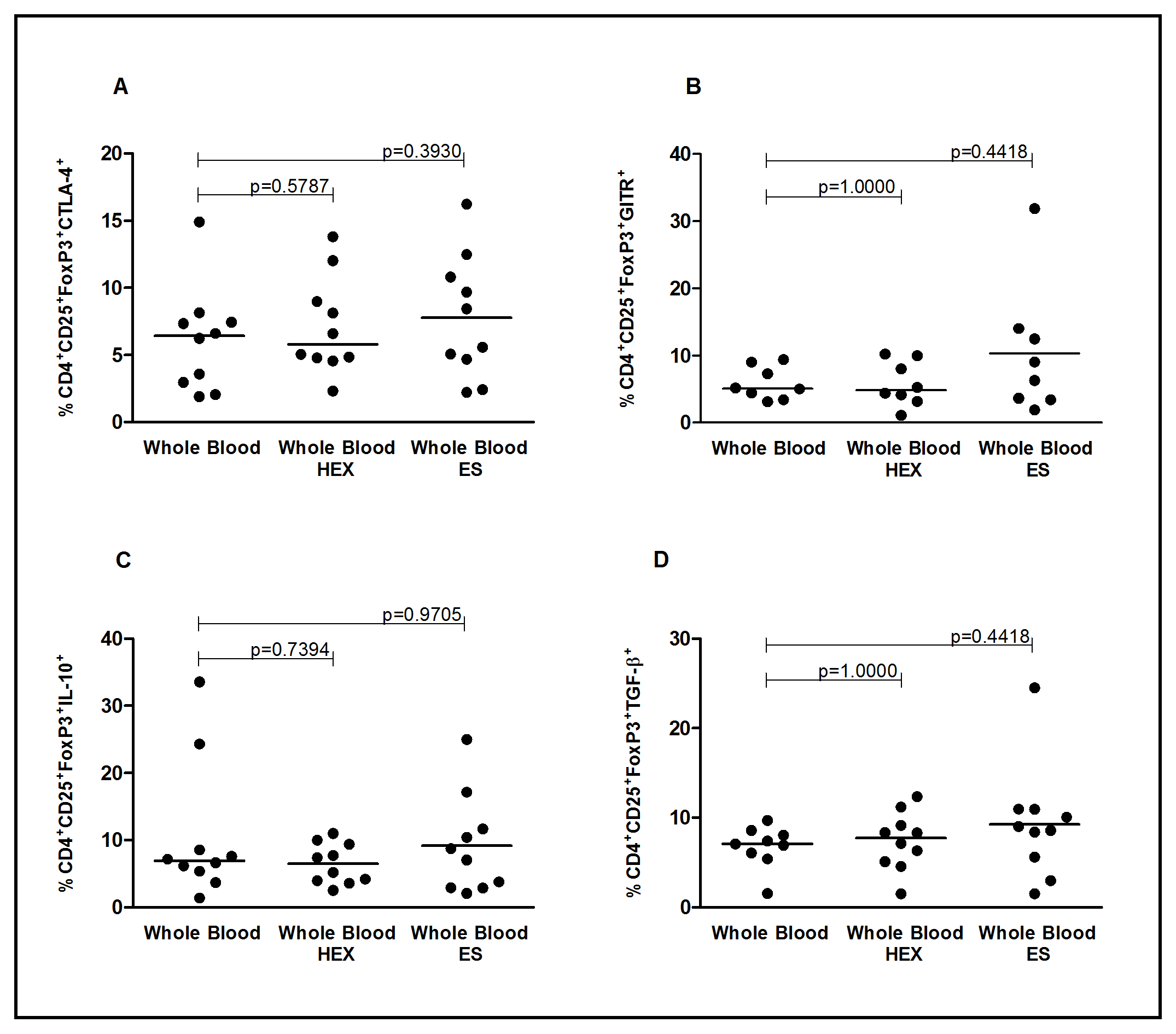

Supplement: Figure S3 — Effect of direct stimulation of whole blood with hookworm antigens in the CD4+CD25+FOXP3+ regulatory T cells producing CTLA-4, GITR, IL-10 and TGF-β. Frequency (%) of (A) CD4+CD25+FOXP3+CTLA-4+, (B) CD4+CD25+FOXP3+GITR+, (C) CD4+CD25+FOXP3+IL-10+ and (D) CD4+CD25+FOXP3+TGF-β+ are indicated on Y-axis. Hookworm adult crude extract (HEX) and excretory-secretory (ES) products were used in the whole blood cultures of Necator-infected and non-infected donors (n = 10 for both groups). (TIF) [file pntd.0001383.s003.tif]

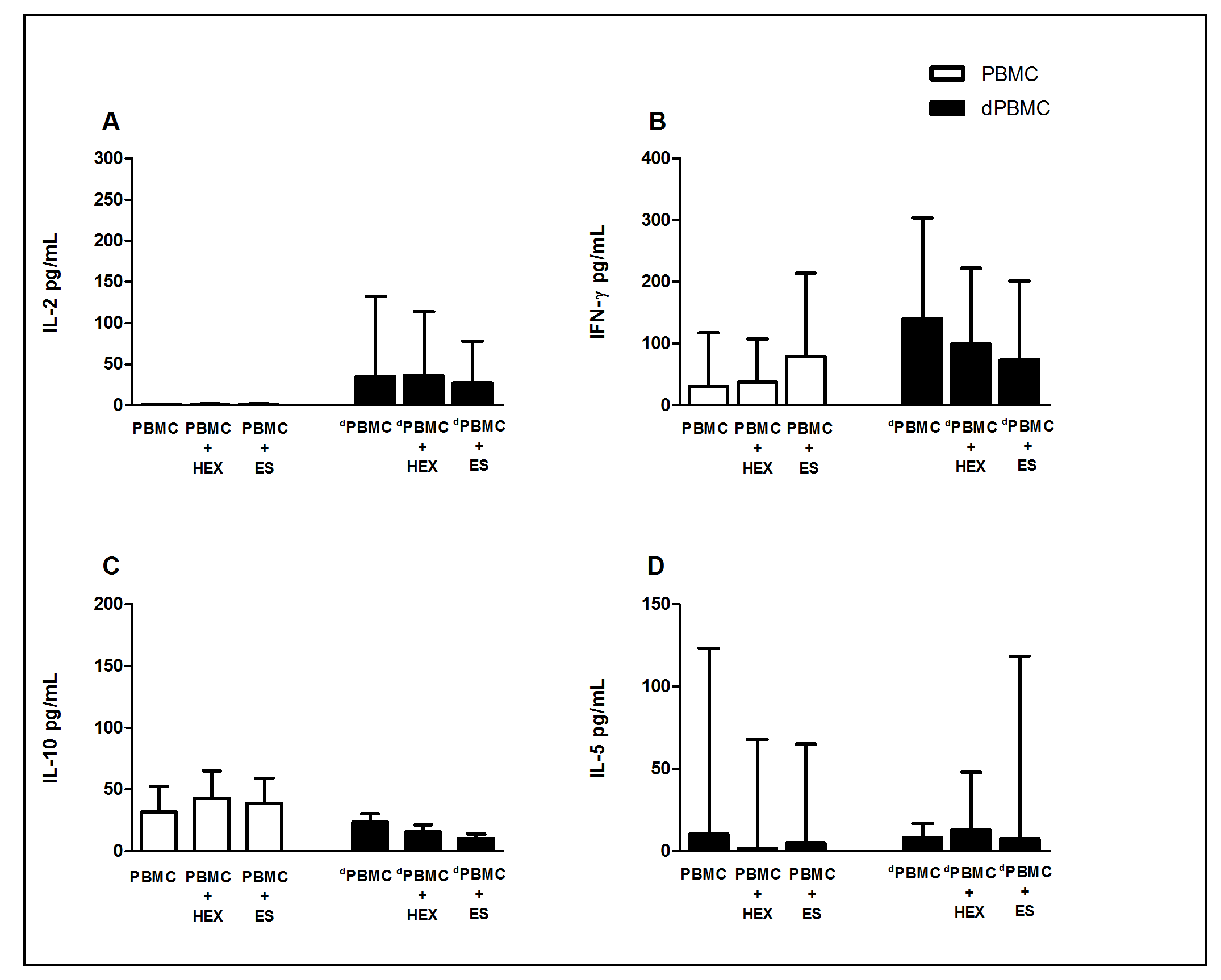

Supplement: Figure S4 — Cytokine concentrations (mean ± standard error) of IL-2 (A), IFN-γ (B), IL-10 (C) and IL-5 (D) in supernatants from cultures (n = 10) of PBMCs and CD4+CD25+-depleted PBMCs (dPBMCs) after stimulation with crude extract (HEX) and excretory-secretory (ES) products. Results are expressed in pg/mL. (TIF) [file pntd.0001383.s004.tif]

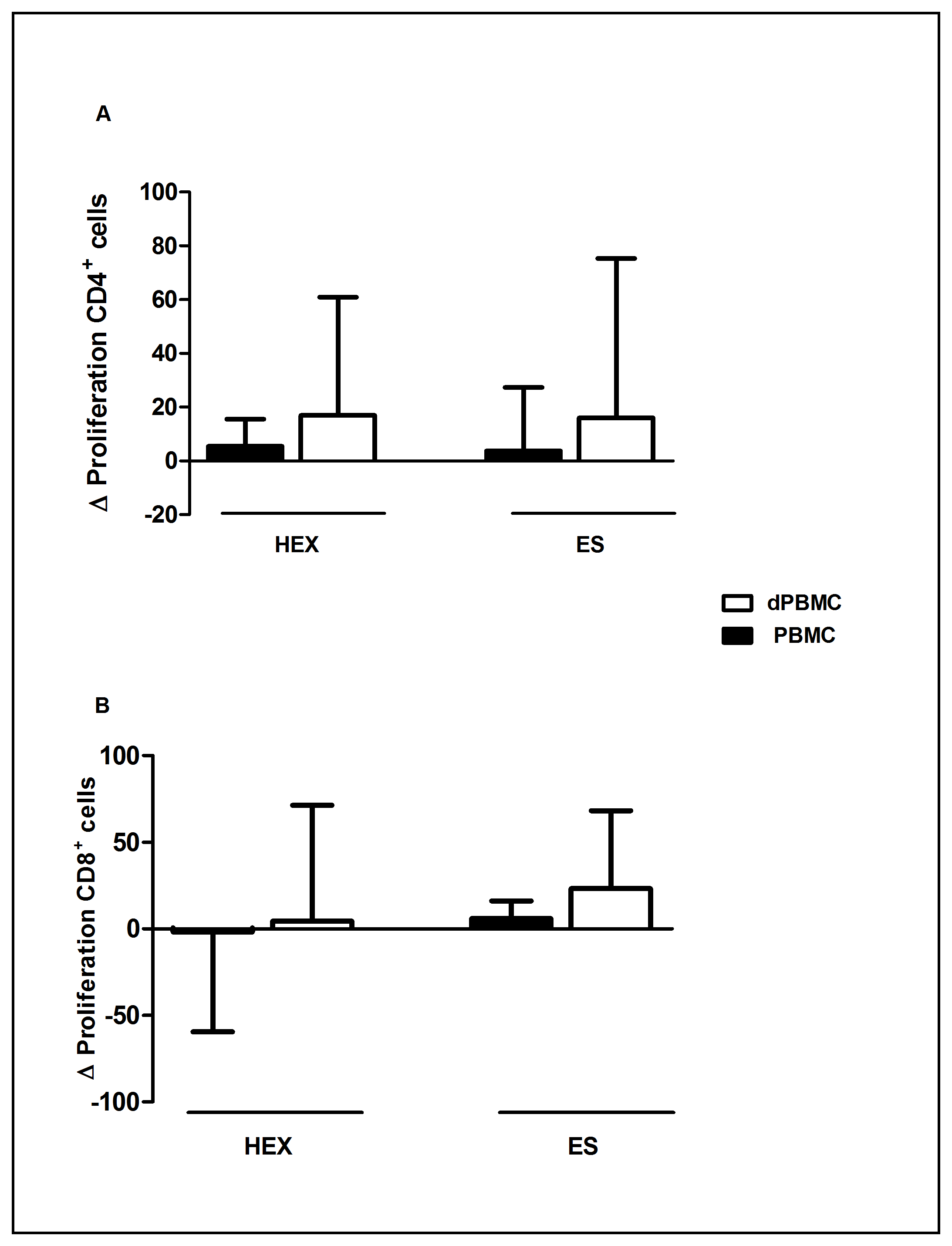

Supplement: Figure S5 — ΔCFDA-SE Proliferation of (A) CD4+ or (B) CD8+ in PBMCs and Treg-depleted PBMCs (dPBMCs) from control egg-negative donors (n = 10) after stimulation with hookworm adult crude extract (HEX) and excretory-secretory (ES) products. ΔCFDA-SE Proliferation was calculated by proliferative response observed in stimulated PBMCs/dPBMCs (indicated by positivity for CFDA-SE) subtracted from basal proliferative response of non-stimulated cells (PBMCs or dPBMCs only. (TIF) [file pntd.0001383.s005.tif]
